# Estimating the Extent of Missed Opportunities for Osteoporosis Treatment Before Hip Fracture Occurrence

**DOI:** 10.1007/s00223-026-01594-8

**Published:** 2026-08-29

**Authors:** Gaetano Paride Arcidiacono, Marco Onofrio Torres, Chiara Ceolin, Mattia Giantin, Giovanni Tripepi, Valentina Camozzi, Anna Bertocco, Mor Peleg Falb, Martin Diogo, Francesca Guidolin, Giacomo Voltan, Maria Grazia Rodà, Elisa Pala, Andrea Angelini, Deris Gianni Boemo, Maria Vittoria Nesoti, Elena Campello, Pietro Ruggieri, Maria Fusaro, Paolo Simioni, Giuseppe Sergi, Sandro Giannini, Marina De Rui, Stefania Sella

**Affiliations:** 1 Department of Medicine, University of Padova, Padova, Italy; 2 Clinica Medica 1, Department of Medicine, Azienda Ospedale-Università Padova, Padova, Italy; 3 Geriatrics Division, Department of Medicine, Azienda Ospedale-Università Padova, Padova, Italy; 4 Institute of Clinical Physiology (IFC), National Research Council (CNR), Reggio Calabria, Italy; 5 Endocrinology Unit, Department of Medicine, Azienda Ospedale-Università Padova, Padova, Italy; 6 Department of Orthopedics and Orthopedic Oncology, D.I.S.C.O.G. Department, University of Padova, Padova, Italy; 7 Department of Directional Hospital Management, Azienda Ospedale-Università Padova, Padova, Italy; 8 National Research Council (CNR), Institute of Clinical Physiology (IFC), Pisa, Italy; 9 Metabolic Bone Disease Unit, Azienda Ospedale-Università Padova, Padova, Italy

**Keywords:** Osteoporosis, Hip fracture, Treatment gap, Fracture risk assessment, Fracture Liaison service, Fracture prevention

## Abstract

Hip fractures (HF) are among the most serious consequences of osteoporosis. Many individuals who subsequently sustain a HF may already fulfill recognized criteria for fracture risk assessment, bone mineral density (BMD) evaluation, or anti-osteoporosis treatment before the event occurs, yet remain unidentified and untreated. We aimed to quantify these missed opportunities for osteoporosis management prior to fragility HF occurrence. We performed a cross-sectional analysis of 1,103 patients aged ≥ 50 years hospitalized for fragility HF and evaluated within a Fracture Liaison Service. Pre-fracture fracture risk was retrospectively estimated using FRAX^®^ based on clinical risk factors only, excluding the index HF. Eligibility for anti-osteoporosis treatment and BMD assessment was evaluated according to international recommendations and national reimbursement criteria. Only 82 patients (7.4%) had received anti-osteoporosis therapy before the index HF. Female sex, rheumatologic diseases, chronic glucocorticoid therapy, and previous fractures were independently associated with prior treatment, whereas diabetes mellitus was negatively associated. Among patients aged 50–90 years, 69.6% would have been classified as being at high or very high fracture risk before fracture occurrence, yet only 10.2% had received anti-osteoporosis therapy. Among the remaining patients, 90.3% already met indications for BMD assessment. Most patients who sustain a HF already meet recognized criteria for osteoporosis assessment or treatment but remain untreated. These findings highlight substantial missed opportunities for fracture prevention and support earlier identification and management of individuals at increased fracture risk.

**Supplementary Information:**

The online version contains supplementary material available at https://doi.org/10.1007/s00223-026-01594-8.

## Introduction

Hip fractures (HFs) are among the most serious consequences of osteoporosis and represent a major clinical and economic burden worldwide. Across the EU6 (France, Germany, Italy, Spain, the UK, and Sweden), approximately 526,000 HFs occurred in 2017, and the annual incidence is projected to increase by 28% by 2030 as a result of population ageing [1]. HFs are associated with high mortality, loss of independence, reduced mobility, and increased need for long-term institutional care [2–4]. Effective antiresorptive and anabolic therapies are now widely available and have proven highly effective in increasing bone mineral density (BMD) and reducing fracture risk [5]. It is known that failure to initiate anti-osteoporosis treatment in high-risk individuals, particularly those with prior fragility fractures, significantly increases the likelihood of subsequent fractures [6]. Nevertheless, a substantial treatment gap persists, with most eligible patients remaining untreated [7]. Fracture Liaison Services (FLS) have been established to improve secondary fracture prevention through systematic identification, assessment, and treatment of patients after a fragility fracture. These programs have been shown to improve post-fracture care (PFC), reducing the risk of subsequent fractures, with a favorable cost-effectiveness profile [8, 9]. While hospital-based PFC models are now considered essential, they primarily address secondary prevention; a considerable proportion of fragility fractures could be prevented before their occurrence through timely identification and treatment of individuals at elevated fracture risk. Tools such as the Fracture Risk Assessment Tool (FRAX^®^) [10], combined with clinical risk factors and BMD assessment, allow recognition of patients who may already qualify for treatment before sustaining a major osteoporotic fracture (MOF). Yet, nearly 75% of high-risk women in European primary care remain untreated [11].

Given these premises, the aim of this study was to estimate the extent of missed opportunities for osteoporosis management before HF occurrence. Specifically, we sought to determine how many patients who subsequently sustained a HF would already have fulfilled recognized indications for anti-osteoporosis treatment or, alternatively, for BMD assessment before the index event. In addition, we characterized the clinical profile of treated and untreated patients and evaluated these opportunities according to both international recommendations and the Italian regulatory framework.

## Patients and Methods

### Study Population

We conducted an observational cohort study including patients aged ≥ 50 years admitted for a fragility HF and evaluated within our FLS program, previously described elsewhere [12]. The study was conducted and reported according to STROBE guidelines [13], and included patients assessed between March 2023 and March 2025. Data were extracted from computerized hospital records and included demographic and anthropometric characteristics, comorbidities (including the Charlson Comorbidity Index or CCI [14]), skeletal fragility risk factors (previous fragility fractures, family history of fractures, smoking, glucocorticoid use, and other relevant conditions), and prior use of vitamin D and anti-osteoporosis medications. BMD data were generally unavailable because Dual-energy X-ray Absorptiometry (DXA) was not routinely performed in the acute post-HF setting, and previous DXA results could not be consistently retrieved.

### Assessment of Fracture Risk

Fracture risk was estimated using the FRAX^®^ tool (Italian version) [15], to calculate 10-year probabilities of MOF and HF, based on clinical risk factors only. As FRAX^®^ is validated for individuals aged up to 90 years, analyses were restricted to patients aged 50–90 years. To retrospectively estimate pre-fracture risk, the index HF was not included in the calculation. Patients were classified as low, intermediate, high, or very high risk according to age-specific National Osteoporosis Guideline Group (NOGG) intervention thresholds [16], which define indications for BMD testing and/or pharmacological treatment. Final risk classification was based on the highest category derived from MOF or HF probability.

### Eligibility for Treatment and BMD Assessment According to International Criteria

To estimate the proportion of patients who would have been eligible for anti-osteoporosis treatment before the index HF, eligibility was assessed according to internationally endorsed osteoporosis management recommendations [10, 16]. Patients were considered treatment-eligible if their pre-fracture FRAX^®^ probability corresponded to a high or very high fracture risk category. We also identified patients who, despite not being classified as high or very high risk, would have met criteria for BMD testing before the index fracture. This group included individuals at intermediate FRAX^®^ risk and those meeting International Society for Clinical Densitometry (ISCD) indications for densitometric assessment [17].

### Reimbursability of Treatment and BMD Assessment According to Italian National Criteria

Given country-specific differences in osteoporosis care pathways, we also evaluated treatment eligibility according to the Italian regulatory framework. Reimbursability of anti-osteoporosis therapies was assessed using Italian Medicines Agency (AIFA) Note 79 [18], which defines the conditions for National Health Service reimbursement. As BMD data were unavailable, only BMD-independent criteria were considered (Table S1). To contextualize fracture risk within Italian clinical practice, risk stratification was also performed using DeFRA, a validated Italian tool estimating 10-year MOF probability in women [19]. DeFRA was calculated in women aged 50–90 years using clinical risk factors only, excluding the index HF, and a ≥ 20% threshold was used to define high fracture risk [20].

Access to reimbursed DXA testing was evaluated according to the Italian “Livelli Essenziali di Assistenza” (LEA) criteria (Table S2), to determine whether patients would have met indications for BMD assessment before the index fracture [21].

### Statistical Analysis

Continuous variables are reported as mean ± SD or median (IQR), according to distribution, and categorical variables as counts and percentages. Normality was assessed using the Shapiro–Wilk test. Group comparisons were performed using Student’s t-test or Mann–Whitney U test for continuous variables and χ² or Fisher’s exact test for categorical variables, as appropriate. Associations between patient characteristics and anti-osteoporosis treatment prior to the index fracture were evaluated using logistic regression. In the overall cohort, univariable analyses were first performed for each variable, followed by age- and sex-adjusted analyses (Model 1); variables applicable only to women were adjusted for age alone. Variables associated with treatment at *p* < 0.10 in univariable analyses, together with prespecified clinically relevant covariates, were considered for inclusion in the multivariable model (Model 2). Covariates were excluded in the presence of multicollinearity, substantial conceptual overlap, or sparse data. To explore potential sex-related differences, descriptive analyses were stratified by sex. Logistic regression analyses were also repeated among women, including univariable models and a multivariable model comprising variables associated with treatment at *p* < 0.10 in univariable analyses. A corresponding regression analysis was not performed among men because of the small number of previously treated male patients, resulting in sparse data and precluding reliable effect estimates. Results are presented as odds ratios (ORs) with 95% confidence intervals (95% CIs).Statistical analyses were performed using Python (version 3.14) with the pandas, NumPy, SciPy, and statsmodels packages. Statistical significance was set at *p* < 0.05.

## Results

### Clinical Characteristics of the Patients

**Fig. 1:**
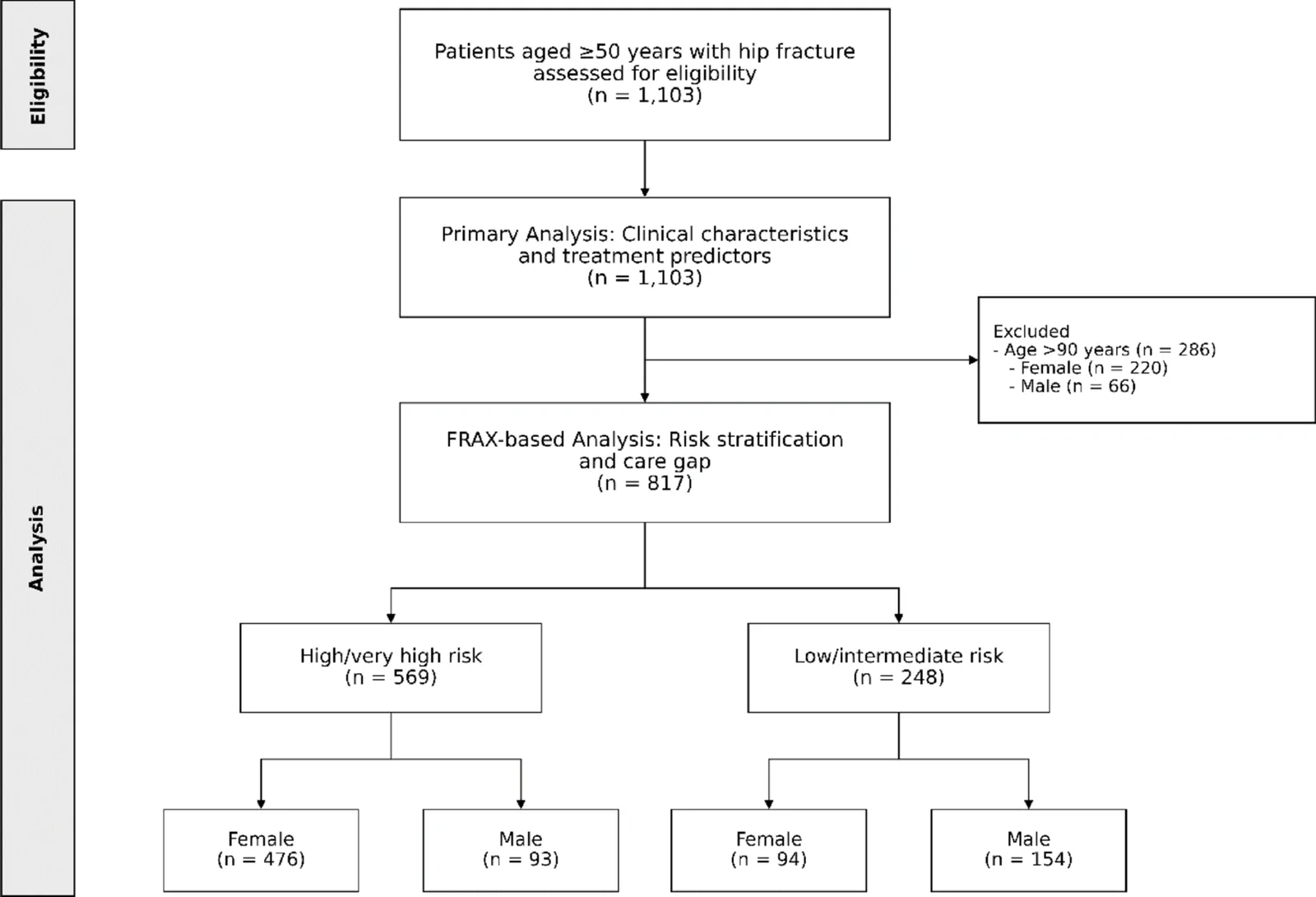
Flow diagram of patient selection and risk stratification. The flowchart illustrates the recruitment and assessment process. Of the 1103 patients aged ≥ 50 years hospitalized for hip fracture and evaluated within the Hip-POS FLS program, 286 individuals were excluded from the FRAX®-based analysis because the tool is not validated for patients aged > 90 years. The remaining 817 patients were stratified using age-specific NOGG intervention thresholds into high or very high risk and low or intermediate risk groups, with the number of women and men reported separately for each risk group

A total of 1,103 patients hospitalized for HF were included (Fig. 1), comprising 790 women (71.6%) and 313 men (28.4%). Overall, 82 patients (7.4%) had received anti-osteoporosis treatment before the index HF, including 75 women and 7 men. Clinical characteristics according to treatment status are presented separately for women and men in Table 1. Among women, treated patients were younger and more frequently had rheumatologic diseases, previous fractures (including previous MOF and vertebral fractures), and chronic glucocorticoid therapy, whereas diabetes mellitus was more frequent among untreated women. DeFRA scores were also higher among treated women. In men, no significant differences according to treatment status were observed, although comparisons were limited by the small number of previously treated patients (*n* = 7). The corresponding analysis in the overall cohort is provided in Table S3. Vitamin D supplementation was more frequent among treated than untreated patients (79.3% vs. 29.8%, *p* < 0.001). Among previously treated patients, oral bisphosphonates were the most frequently prescribed drugs (*n* = 49), followed by zoledronic acid (*n* = 22), denosumab (*n* = 6), teriparatide (*n* = 4), and romosozumab (*n* = 1).

### Predictors of Receiving Anti-Osteoporosis Treatment Before the Index Fracture

Factors associated with prior anti-osteoporosis treatment were explored using logistic regression (Table 2). Female sex, rheumatologic diseases, chronic glucocorticoid therapy, previous fractures, and higher FRAX^®^ and DeFRA scores were associated with a greater likelihood of treatment, whereas diabetes mellitus was associated with lower odds. These associations were largely confirmed after age- and sex-adjustment (Model 1), while female sex, rheumatologic diseases, chronic glucocorticoid therapy, previous fractures, and diabetes mellitus remained independently associated with prior treatment in the multivariable model (Model 2).

To further explore potential sex-related differences, additional sex-stratified analyses were performed. Among women, diabetes mellitus remained independently associated with lower odds of treatment, whereas rheumatologic diseases, previous fractures, and chronic glucocorticoid therapy were associated with greater odds (Table S4). Age and previous myocardial infarction were not independently associated with treatment in the female-specific multivariable model. A corresponding regression analysis was not performed among men because the small number of previously treated male patients.

### Opportunities for Osteoporosis Treatment Before Index HF

**Fig. 2:**
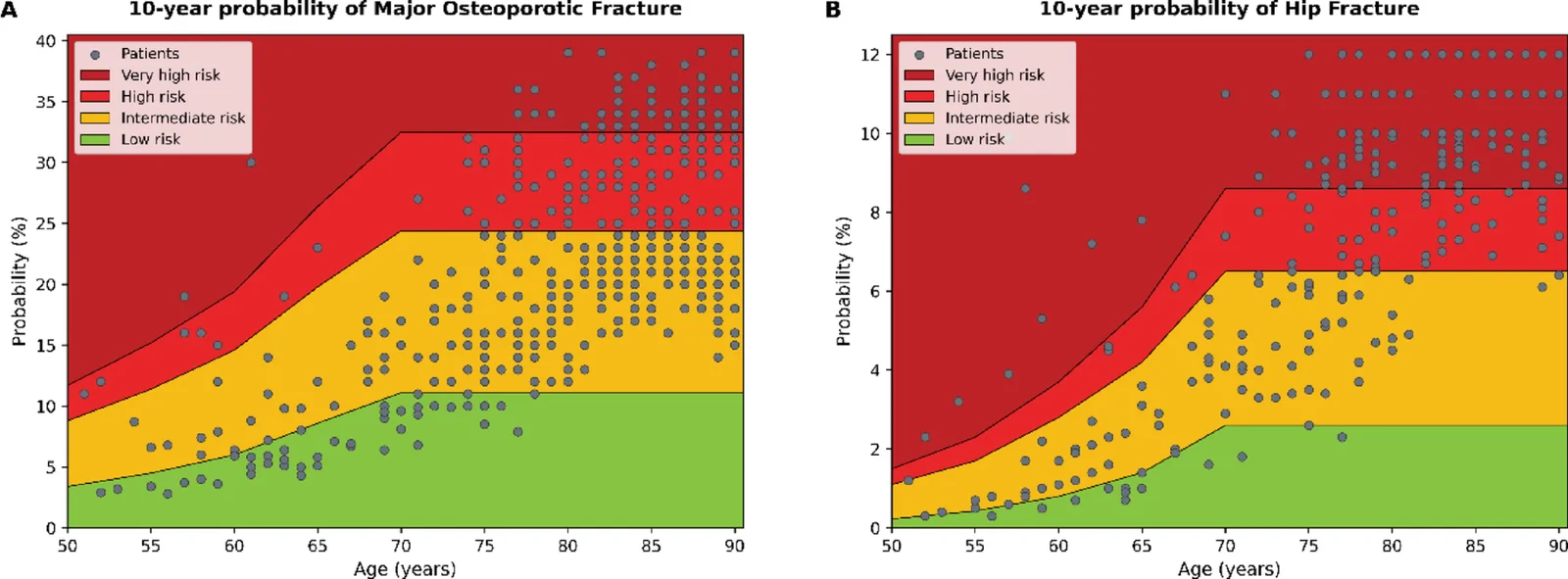
FRAX^®^-derived 10-year fracture probabilities in female subjects (*n* = 570). The left and right panels show, respectively, the FRAX^®^-derived 10-year probability of major osteoporotic fracture and hip fracture. Colored areas represent age-specific FRAX^®^ (Fracture Risk Assessment Tool) intervention thresholds defining low, intermediate, high, and very high fracture risk categories. Grey dots represent individual patients

**Fig. 3:**
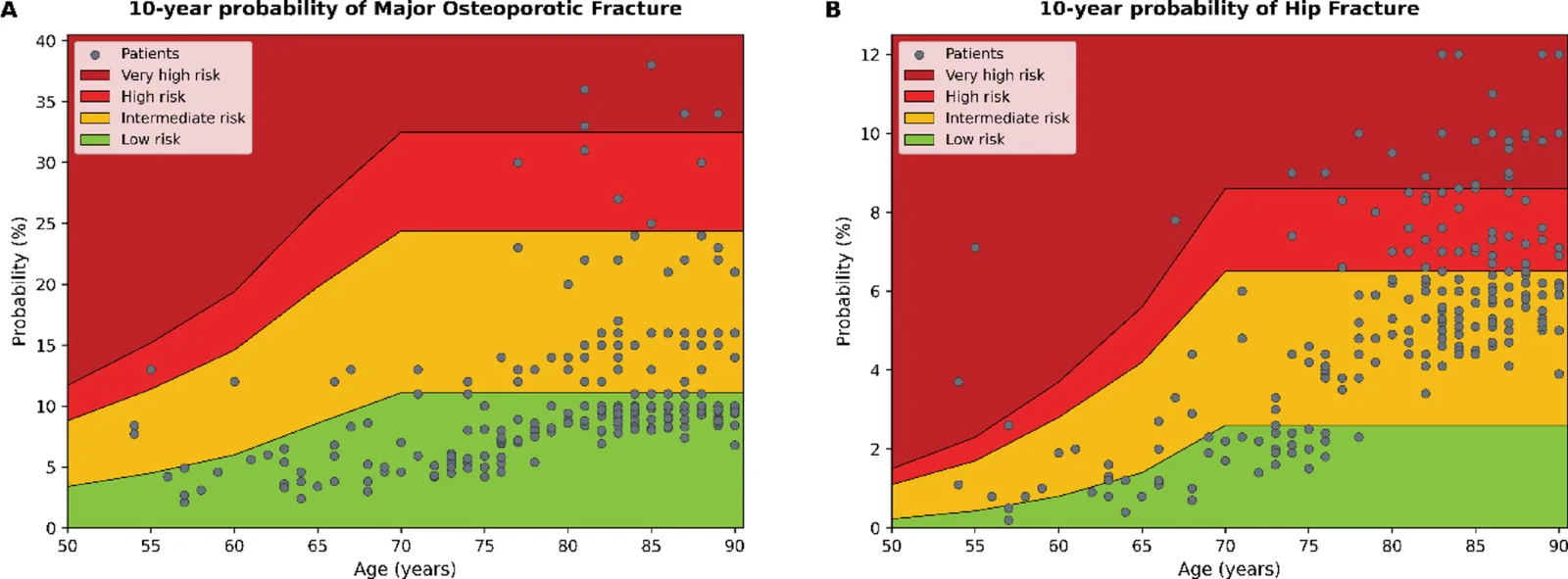
FRAX^®^-derived 10-year fracture probabilities in male subjects (*n* = 247). The left and right panels show, respectively, the FRAX^®^-derived 10-year probability of major osteoporotic fracture and hip fracture. Colored areas represent age-specific FRAX^®^ (Fracture Risk Assessment Tool) intervention thresholds defining low, intermediate, high, and very high fracture risk categories. Grey dots represent individual patients

To quantify missed opportunities for anti-osteoporosis treatment before the index HF, patients were stratified according to the highest FRAX^®^-derived MOF or HF probability. Overall, risk was classified as low in 7.1%, intermediate in 23.3%, high in 10.8%, and very high in 58.9% of patients. Risk distribution differed significantly by sex (*p* < 0.001), with women more frequently classified in the higher-risk categories (Fig. 1). HF probability resulted in a higher risk category than MOF probability in 71.6% of patients, whereas MOF probability resulted in a higher category in only one patient; both probabilities yielded the same risk category in 28.3% of cases. FRAX^®^-derived MOF and HF probabilities are illustrated separately for women in Fig. 2 and men in Fig. 3.

As current guidelines recommend initiating anti-osteoporosis therapy in individuals at high or very high fracture risk, patients classified as high or very high risk were compared with those at low or intermediate risk, as reported in Table S5. Despite their elevated fracture risk, only 10.2% of patients at high or very high risk had received anti-osteoporosis treatment before the index HF. Within this high/very high-risk group, prior treatment was less frequent in men than in women (4.3% vs. 11.3%, *p* = 0.039).

### Opportunities for Osteoporosis Assessment Before Index HF

**Fig. 4:**
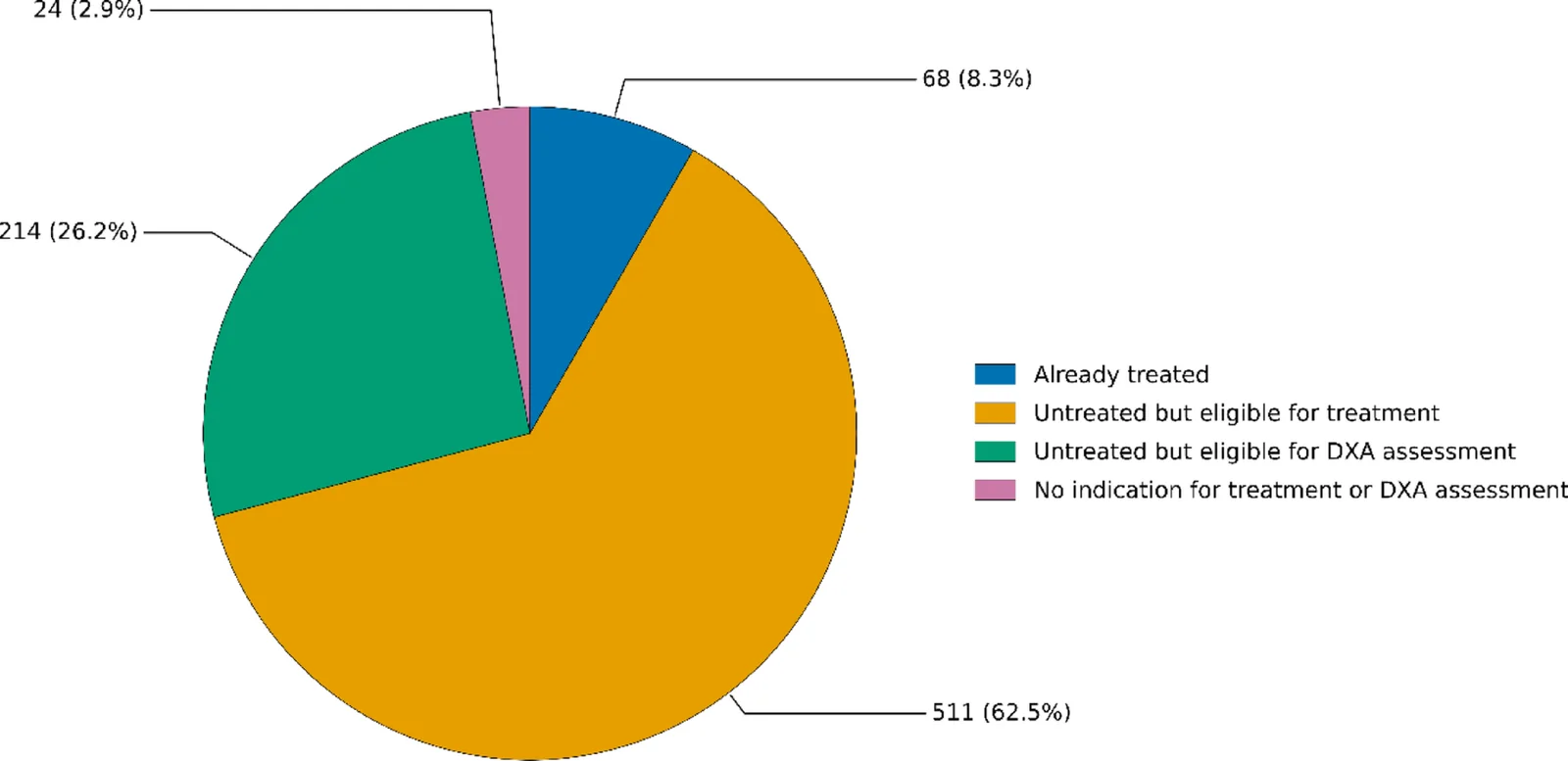
Pre-fracture opportunities for osteoporosis management. Pie chart showing mutually exclusive categories of osteoporosis management before the index HF in patients aged 50–90 years: already treated before the index HF; eligible for treatment without DXA assessment (i.e., FRAX^®^ high or very high risk) but untreated; eligible for BMD assessment (FRAX^®^ intermediate risk and/or fulfillment of ISCD criteria); and no indication for treatment or assessment. *Abbreviations*: BMD, bone mineral density; DXA, dual-energy X-ray absorptiometry; HF, hip fracture; ISCD, International Society for Clinical Densitometry

After excluding the 569 patients classified as high or very high fracture risk, who already met indications for anti-osteoporosis treatment, we evaluated the remaining 248 patients for potential indications for BMD testing before the index HF. Among them, 190 were classified as intermediate risk according to FRAX^®^ and 210 fulfilled ISCD criteria for BMD testing. Overall, 224 patients (90.3%) met at least one indication for osteoporosis assessment, either through intermediate FRAX^®^ risk or ISCD criteria, whereas only 24 (9.7%) had no indication for further evaluation (Fig. 4). These 24 patients were predominantly male (66.7%), relatively young (mean age 62.1 ± 4.9 years), had a mean BMI of 26.4 kg/m^2^ and a low comorbidity burden (median CCI 2). They lacked classical osteoporosis-related risk factors and did not meet FRAX^®^- or ISCD-based criteria for osteoporosis assessment or treatment.

### Opportunities for Osteoporosis Treatment And Assessment Before Index HF According to Italian Regulations

Beyond the gaps identified according to international recommendations, osteoporosis care was also evaluated according to Italian regulations. Among the 570 women in whom DeFRA was available, 295 (51.8%) were classified as being at high fracture risk and therefore eligible for treatment; only 15.6% of these women had received prior anti-osteoporosis therapy. In the overall cohort, including both women and men, 208 patients (18.9%) would have been eligible for anti-osteoporosis therapy reimbursed by the Italian National Health Service (SSN) under AIFA Note 79 (Table S1), based on clinical criteria alone and without requiring BMD assessment. Among the remaining 895 patients, 366 (40.9%) met the Italian “Livelli Essenziali di Assistenza” (LEA) criteria for reimbursed BMD assessment (Table S2).

## Discussion

This study shows that most patients who eventually sustained a HF could already have been identified as candidates for osteoporosis treatment or further diagnostic assessment before the fracture occurred. In particular, only 7.4% of patients hospitalized for HF had received anti-osteoporosis therapy before the index event, confirming a substantial osteoporosis care gap despite the availability of validated fracture risk assessment tools and effective treatments. This finding is consistent with previous reports showing that only 15.0–18.3% of high- and very high-risk patients receive treatment before sustaining a fragility fracture [22]. Sex-stratified analyses highlighted differences in osteoporosis management between women and men. Although women were more frequently classified as being at high or very high fracture risk, prior treatment remained uncommon in both sexes and was less frequent among men within the high/very high-risk group. This finding may reflect the persistent perception of osteoporosis as predominantly a female disease, lower awareness of skeletal fragility in men, and less frequent risk assessment or referral for diagnostic evaluation [23]. Rheumatologic diseases and chronic glucocorticoid therapy were associated with a higher likelihood of prior treatment, likely because these conditions are widely recognized as fracture risk factors. In contrast, diabetes mellitus was associated with lower treatment rates, possibly reflecting reduced awareness of fracture risk and the frequent presence of normal or near-normal BMD despite increased fracture risk [24–26]. The low use of vitamin D supplementation among untreated patients further suggests limited recognition and management of skeletal health before HF occurrence, particularly given the high prevalence of vitamin D deficiency among older adults with HF [27].

Beyond quantifying the proportion of patients who had already received anti-osteoporosis therapy and identifying the clinical profile of treated and untreated patients, we also estimated how many patients would already have met current indications for pharmacological treatment or further osteoporosis assessment before the index fracture. Overall, 69.6% of patients would have been classified as being at high or very high fracture risk according to FRAX^®^ before HF occurrence. Yet, only 10.2% of patients at high or very high risk had received anti-osteoporosis treatment. Furthermore, when patients at intermediate FRAX^®^ risk and/or meeting ISCD criteria for DXA were also considered, nearly all patients would already have met criteria for either treatment or further osteoporosis assessment. Thus, the missed opportunity extended beyond failure to initiate treatment to failure to identify individuals who already warranted further diagnostic evaluation.

Our analysis was not intended to estimate how many HFs could have been prevented with earlier pharmacological therapy, as such an assessment would require longitudinal data beyond the scope of this study. Nevertheless, randomized trials show that anti-osteoporotic medications reduce the risk of MOF and HF by approximately 30–70%, depending on the drug and fracture type [28]. Given that many patients in our cohort had already met recognized indications for treatment, earlier intervention may have prevented at least some of these events.

Several factors may contribute to the osteoporosis care gap, affecting both fracture risk assessment and treatment initiation, including limited awareness of osteoporosis among patients and healthcare professionals, inconsistent use of fracture risk assessment tools, competing clinical priorities, disability, reduced physical performance, and osteoarticular conditions in older adults, as well as organizational barriers and inequalities in access to diagnostic services [29–31].

Within the Italian healthcare system, the osteoporosis treatment gap has been estimated at approximately 71% [32], with some FLS cohorts reporting pre-fracture treatment gaps exceeding 96% [33, 34]. This may partly reflect regulatory barriers, as reimbursement for anti-osteoporosis therapies and DXA examinations is governed by AIFA Note 79 and LEA criteria, which may delay access to treatment and diagnostic evaluation compared with international recommendations [35].

Some limitations of this study should be acknowledged. First, this was a single-centre study conducted in a tertiary hospital setting, which may limit the generalizability of the findings to other healthcare systems or organizational contexts. Second, the analysis included only patients who sustained HF, potentially capturing individuals who had not received adequate osteoporosis management, as patients effectively treated for osteoporosis may have been less likely to experience a fracture. The lack of pre-fracture DXA data prevented evaluation of BMD-based eligibility for treatment and may have led to an underestimation of potential treatment opportunities. In addition, the limited number of previously treated men precluded more detailed sex-specific analyses. Finally, some regression estimates had wide confidence intervals, reflecting limited precision due to the small number of events. Despite these limitations, this study has several strengths. It analyzes a large, well-characterized real-world cohort of patients who sustained a HF, providing clinically relevant insights into gaps in osteoporosis care prior to the fracture event. Fracture risk was retrospectively estimated using pre–index fracture characteristics, allowing a realistic assessment of patients who would have met criteria for fracture risk assessment or anti-osteoporosis treatment before the event. Moreover, by integrating international guideline recommendations with national regulatory criteria, our analysis provides a comprehensive overview of missed opportunities for osteoporosis assessment and treatment within different decision-making frameworks.

In conclusion, our study shows that most patients who subsequently sustained a HF would already have met recognized criteria for osteoporosis treatment or further diagnostic assessment before the index event, yet only a small minority had received anti-osteoporosis therapy. These findings suggest that substantial opportunities for fracture prevention are missed before the first HF occurs, despite the availability of validated fracture risk assessment tools and effective treatments. Earlier identification of individuals at increased fracture risk and timely implementation of appropriate diagnostic and therapeutic strategies may help reduce the burden of fragility fractures in clinical practice.

**Table 1:** Clinical characteristics of patients according to anti-osteoporosis treatment prior to the index hip fracture, stratified by sex

Variables	Female subjects (*n* = 790)			Male subjects (*n* = 313)		
	Untreated (*n* = 715)	Treated (*n* = 75)	*p*	Untreated (*n* = 306)	Treated (*n* = 7)	*p*
Age (years), mean (SD)	83.4 (9.2)	80.3 (7.5)	**0.001**	81.8 (9.3)	85.7 (5.7)	0.127
BMI (Kg/m^2^), mean (SD)	23.8 (4.5)	23.6 (4.5)	0.703	24.9 (3.9)	23.1 (2.8)	0.146
*Comorbidities*						
Diabetes mellitus, n (%)	120 (16.8)	4 (5.3)	**0.010**	67 (21.9)	0 (0.0)	0.353
History of myocardial infarction, n (%)	60 (8.4)	2 (2.7)	0.079	61 (19.9)	1 (14.3)	1.000
Heart failure, n (%)	67 (9.4)	6 (8.0)	0.697	38 (12.4)	0 (0.0)	1.000
History of stroke, n (%)	51 (7.1)	4 (5.3)	0.560	42 (13.7)	1 (14.3)	1.000
COPD, n (%)	29 (4.1)	6 (8.0)	0.133	43 (14.1)	0 (0.0)	0.599
CKD stage IV-V, n (%)	22 (3.1)	3 (4.0)	0.724	18 (5.9)	0 (0.0)	1.000
Liver cirrhosis, n (%)	1 (0.1)	3 (4.0)	**0.003**	7 (2.3)	0 (0.0)	1.000
Rheumatologic diseases, n (%)	33 (4.6)	12 (16.0)	**< 0.001**	11 (3.6)	0 (0.0)	1.000
Dementia, n (%)	168 (23.5)	13 (17.3)	0.227	78 (25.5)	1 (14.3)	0.684
CCI, median (IQR)	5 (4–6)	5 (4–6)	0.986	6 (4–7)	6 (4–8)	0.906
*Risk factors for skeletal fragility*						
Previous fracture, n (%)	229 (32.0)	35 (46.7)	**0.011**	69 (22.5)	2 (28.6)	0.659
Previous MOF, n (%)	169 (23.6)	26 (34.7)	**0.035**	44 (14.4)	2 (28.6)	0.274
Previous HF, n (%)	45 (6.3)	2 (2.7)	0.303	15 (4.9)	1 (14.3)	0.310
Previous vertebral fracture, n (%)	67 (9.4)	19 (25.3)	**< 0.001**	23 (7.5)	1 (14.3)	0.431
Previous humerus fracture, n (%)	33 (4.6)	3 (4.0)	1.000	5 (1.6)	1 (14.3)	0.128
Previous wrist fracture, n (%)	56 (7.8)	5 (6.7)	0.719	9 (2.9)	0 (0.0)	1.000
Previous fracture at another site, n (%)	104 (14.5)	12 (16.0)	0.735	33 (10.8)	0 (0.0)	1.000
Family history of hip fracture, n (%)	68 (9.5)	8 (10.7)	0.747	34 (11.1)	0 (0.0)	1.000
Active smoking, n (%)	38 (5.3)	7 (9.3)	0.183	37 (12.1)	1 (14.3)	0.601
Chronic glucocorticoid therapy, n (%)	10 (1.4)	9 (12.0)	**< 0.001**	7 (2.3)	0 (0.0)	1.000
FRAX^®^ MOF, median (IQR)^†^	22.0 (17.8–30.0)	22.5 (18.0–33.0)	0.154	9.6 (7.4–13.0)	10.0 (9.1–13.8)	0.307
FRAX^®^ HF, median (IQR)^†^	12.0 (8.3–17.0)	13.0 (8.8–21.2)	0.169	5.6 (3.8–7.6)	7.0 (6.2–8.1)	0.168
DeFRA, median (IQR)^‡^	19.0 (14.0–31.0)	29.0 (19.2–51.8)	**< 0.001**	–	–	–

^†^Referring only to subjects aged 50–90 years. ^‡^Referring only to female subjects aged 50–90 years. Statistically significant *p*-values (*p* < 0.05) are highlighted in bold

BMI: body mass index, CCI: Charlson Comorbidity Index, CKD: chronic kidney disease, COPD: chronic obstructive pulmonary disease, DeFRA: Derived Fracture Risk Assessment Tool, FRAX^®^: Fracture Risk Assessment Tool, HF: hip fracture, IQR: interquartile range, MOF: major osteoporotic fracture, SD: standard deviation

**Table 2:** Univariable and multivariable logistic regression analyses of factors associated with anti-osteoporosis treatment prior to the index hip fracture

Variables	Unadjusted		Model 1^a^		Model 2^b^	
	OR (95% CI)	*p*	OR (95% CI)	*p*	OR (95% CI)	*p*
Age (years)	0.98 (0.95–0.99)	**0.040**	0.97 (0.95–0.99)	**0.020**	0.98 (0.96–1.00)	0.109
Female sex	4.59 (2.09–10.06)	**< 0.001**	4.76 (2.17–10.47)	**< 0.001**	4.27 (1.98–9.52)	**< 0.001**
BMI (Kg/m^2^)	0.97 (0.91–1.02)	0.245	0.98 (0.93–1.03)	0.461	–	–
*Comorbidities*						
Diabetes mellitus	0.23 (0.08–0.63)	**0.004**	0.25 (0.09–0.69)	**0.007**	0.24 (0.09–0.68)	**0.007**
History of myocardial infarction	0.28 (0.09–0.91)	**0.034**	0.39 (0.12–1.25)	0.113	–	–
Heart failure	0.69 (0.29–1.62)	0.393	0.84 (0.35–2.00)	0.689	–	–
History of stroke	0.65 (0.26–1.64)	0.360	0.85 (0.33–2.17)	0.727	–	–
COPD	1.04 (0.44–2.47)	0.928	1.55 (0.63–3.77)	0.338	–	–
CKD stage IV-V	0.93 (0.28–3.08)	0.907	1.23 (0.36–4.15)	0.737	–	–
Liver cirrhosis	4.81 (1.25–18.48)	**0.022**	7.24 (1.63–32.23)	**0.009**	–	–
Rheumatologic diseases	3.81 (1.92–7.53)	**< 0.001**	3.30 (1.64–6.64)	**0.001**	2.99 (1.42–6.29)	**0.004**
Dementia	0.65 (0.36–1.17)	0.152	0.75 (0.41–1.39)	0.365	–	–
CCI	0.96 (0.85–1.07)	0.443	1.10 (0.97–1.25)	0.154	–	–
*Risk factors for skeletal fragility*						
Previous fracture	1.99 (1.27–3.15)	**0.003**	1.86 (1.17–2.95)	**0.008**	1.74 (1.08–2.80)	**0.022**
Previous MOF	1.97 (1.22–3.18)	**0.006**	1.81 (1.11–2.95)	**0.017**	–	–
Previous HF	0.60 (0.19–1.86)	0.376	0.59 (0.19–1.87)	0.373	–	–
Previous vertebral fracture	3.34 (1.93–5.78)	**< 0.001**	3.29 (1.88–5.75)	**< 0.001**	–	–
Previous humerus fracture	1.33 (0.46–3.81)	0.600	1.20 (0.41–3.47)	0.741	–	–
Previous wrist fracture	0.96 (0.37–2.44)	0.923	0.81 (0.32–2.10)	0.670	–	–
Previous fracture at another site	1.11 (0.58–2.09)	0.757	1.03 (0.54–1.96)	0.936	–	–
Family history of hip fracture	0.97 (0.46–2.08)	0.943	0.87 (0.40–1.89)	0.717	–	–
Active smoking	1.36 (0.63–2.93)	0.429	1.40 (0.62–3.16)	0.416	–	–
Chronic glucocorticoid therapy	7.27 (3.13–16.89)	**< 0.001**	6.67 (2.74–16.27)	**< 0.001**	5.09 (2.00–12.93)	**0.001**
FRAX^®^ MOF^†^	1.04 (1.02–1.06)	**< 0.001**	1.04 (1.01–1.06)	**0.002**	–	–
FRAX^®^ HF^†^	1.03 (1.01–1.05)	**0.001**	1.03 (1.01–1.05)	**0.010**	–	–
DeFRA^‡^	1.02 (1.01–1.03)	**0.001**	1.02 (1.01–1.03)	**< 0.001**	–	–

^a^Model 1: logistic regression adjusted for age and sex; age and sex were mutually adjusted for each other, while variables applicable only to female subjects were adjusted for age only. ^b^Model 2: multivariable logistic regression model including age, sex, diabetes mellitus, rheumatologic diseases, chronic glucocorticoid therapy, and previous fractures (any site).

^†^Referring only to subjects aged 50–90 years. ^‡^Referring only to female subjects aged 50–90 years. Statistically significant p-values (*p* < 0.05) are highlighted in bold

BMI: body mass index, CCI: Charlson Comorbidity Index, CKD: chronic kidney disease, CI: confidence interval, COPD: chronic obstructive pulmonary disease, DeFRA: Derived Fracture Risk Assessment Tool, FRAX^®^: Fracture Risk Assessment Tool, HF: hip fracture, MOF: major osteoporotic fracture, OR: odds ratio

## Supplementary Information

Below is the link to the electronic supplementary material.


Supplementary Material 1


## Data Availability

The datasets analyzed during the current study are available from the corresponding author on reasonable request. Declarations.
