# Supplementary material for: Estimating the Extent of Missed Opportunities for Osteoporosis Treatment Before Hip Fracture Occurrence
Source: Calcif Tissue Int. 2026 Aug 29;117(1):133. doi: 10.1007/s00223-026-01594-8 (PMC13525934; doi:10.1007/s00223-026-01594-8)
Supplement: Supplementary file 1 [file 223_2026_1594_MOESM1_ESM.pdf]

Estimating the Extent of Missed Opportunities for Osteoporosis Treatment Before Hip Fracture Occurrence

Supplementary Material

Summary

Table S1. Anti-osteoporosis therapy reimbursability criteria according to Italian AIFA (Italian Medicines Agency) Note 79. 2

Table S2. Clinical indications for reimbursed DXA testing according to the Italian "Livelli Essenziali di Assistenza" (LEA). 4

Table S3. Clinical characteristics of patients according to anti-osteoporosis treatment prior to the index hip fracture in the overall cohort. 5

Table S4. Univariable and multivariable logistic regression analyses of factors associated with anti-osteoporosis treatment prior to the index hip fracture among female subjects. 7

Table S5. Clinical characteristics of patients according to FRAX® risk category prior to the index fracture. 9

<table>
<tr><td colspan="4">Table S1. Anti-osteoporosis therapy reimbursability criteria according to Italian AIFA (Italian Medicines Agency) Note 79.</td></tr>
<tr><th colspan="4">Secondary prevention in subjects with previous vertebral or femoral fractures</th></tr>
<tr><th rowspan="2">Condition</th><th colspan="3">Treatment</th></tr>
<tr><th>First choice</th><th>Second choice</th><th>Third choice</th></tr>
<tr><td>1–2 fractures</td><td>Alendronate
Risedronate
Zoledronate</td><td>Denosumab
Ibandronate
Raloxifene
Bazedoxifene</td><td></td></tr>
<tr><td>≥3 fractures</td><td rowspan="4">Teriparatide</td><td rowspan="6">Denosumab
Zoledronate</td><td rowspan="6">Alendronate
Risedronate
Ibandronate</td></tr>
<tr><td>≥1 fracture + lumbar spine or femoral T-score ≤−4</td></tr>
<tr><td>≥1 fracture + treatment >12 months with prednisone or equivalents ≥5 mg/day</td></tr>
<tr><td>New vertebral or femoral fracture despite treatment under Note 79 for at least 1 year</td></tr>
<tr><td>Postmenopausal female patients with lumbar spine or femoral T-score <−2.5 and >−5.0 (T-score <−2.0 and >−5.0 if age >65 years)
+ ≥2 mild vertebral fractures or at least 1 moderate fracture or history of fracture of forearm, humerus, sacrum, pelvis, hip, femur, or tibia in the last 5 years</td><td>Abaloparatide</td></tr>
<tr><td>Female patients with lumbar spine or femoral T-score <−2.5 (<−2.0 if ≥2 moderate or severe vertebral fractures or femoral fracture in the previous 2 years)
+ history of ≥1 moderate or severe vertebral fracture, or ≥2 mild vertebral fractures, or femoral fracture
+ 10-year fracture risk (calculated with a validated risk calculator) high ≥20%
+ inability to follow other effective treatments</td><td>Romosozumab</td></tr>
<tr><th colspan="4">Secondary prevention in subjects with previous non-vertebral and non-femoral fractures</th></tr>
<tr><th rowspan="2">Condition</th><th colspan="3">Treatment</th></tr>
<tr><th>First choice</th><th>Second choice</th><th>Third choice</th></tr>
<tr><td>Lumbar spine or femoral T-score ≤−3</td><td>Alendronate
Risedronate
Zoledronate</td><td>Denosumab
Ibandronate
Raloxifene
Bazedoxifene</td><td></td></tr>
<tr><td>Female patients with lumbar spine or femoral T-score <−2.5
+ history of ≥2 non-vertebral fractures
+ 10-year fracture risk (calculated with a validated risk calculator) high ≥20%
+ inability to follow other effective treatments</td><td>Romosozumab</td><td></td><td></td></tr>
</table>

<table>
<tr><th colspan="4">Primary prevention in postmenopausal women or men aged ≥50 years at high fracture risk due to at least one of the following conditions</th></tr>
<tr><th rowspan="2">Condition</th><th colspan="3">Treatment</th></tr>
<tr><th>First choice</th><th>Second choice</th><th>Third choice</th></tr>
<tr><td>Current or planned treatment for >3 months with prednisone equivalent ≥5 mg/day</td><td>Alendronate
Risedronate
Zoledronate</td><td>Denosumab</td><td></td></tr>
<tr><td>Ongoing adjuvant hormone-blocking therapy in women with breast cancer or men with prostate cancer</td><td>Alendronate
Risedronate
Zoledronate
Denosumab</td><td></td><td></td></tr>
<tr><td>Lumbar spine or femoral T-score ≤−4</td><td rowspan="2">Alendronate
Risedronate</td><td rowspan="2">Denosumab
Zoledronate
Ibandronate
Raloxifene
Bazedoxifene</td><td></td></tr>
<tr><td>Lumbar spine or femoral T-score ≤−3 + at least one of the following conditions:
1) Family history of vertebral or femoral fractures;
2) Comorbidities associated with fracture risk (rheumatoid arthritis or other connective tissue diseases, diabetes, COPD, chronic inflammatory bowel disease, AIDS, Parkinson’s disease, multiple sclerosis, severe motor disability)</td><td></td></tr>
</table>

The highlighted conditions allow reimbursement of anti-osteoporosis therapies irrespective of bone mineral density. *Abbreviations*: AIDS: acquired immunodeficiency syndrome, COPD: chronic obstructive pulmonary disease.

**Table S2. Clinical indications for reimbursed DXA testing according to the Italian “Livelli Essenziali di Assistenza” (LEA).**
**Major risk factors** (at least one of the following)
1. In subjects of any age, both females and males:<br>a. Previous fragility fractures (caused by minimal trauma) or radiological evidence of vertebral fractures.<br>b. Radiological evidence of osteoporosis.<br>c. Chronic therapies (ongoing or planned):<br>Systemic corticosteroids (for more than 3 months at doses ≥5 mg/day of prednisone equivalent).<br>Levothyroxine (at suppressive doses)<br>Antiepileptic drugs<br>Anticoagulants (heparin)<br>Immunosuppressive agents<br>Antiretroviral drugs<br>Lithium salts<br>GnRH agonists<br>Chemotherapy in pediatric age<br>Radiotherapy in pediatric age<br>d. Diseases associated with an increased risk of osteoporosis:<br>Endocrine diseases with significant skeletal involvement (untreated primary amenorrhea, secondary amenorrhea lasting more than one year, hypogonadism, hyperparathyroidism, hyperthyroidism, Cushing’s syndrome, acromegaly, growth hormone deficiency, hyperprolactinemia, type 1 diabetes mellitus).<br>Rickets/osteomalacia.<br>Malnutrition syndromes, including anorexia nervosa and related conditions.<br>Celiac disease and malabsorption syndromes.<br>Severe chronic inflammatory bowel diseases.<br>Chronic cholestatic liver diseases.<br>Cystic fibrosis.<br>Chronic kidney disease, nephrotic syndrome, chronic tubulopathies, and idiopathic hypercalciuria.<br>Hematologic disorders with significant skeletal involvement (multiple myeloma, lymphoma, leukemia, thalassemia, sickle cell disease, mastocytosis).<br>Rheumatoid arthritis (including Still’s disease), ankylosing spondylitis, psoriatic arthritis, systemic connective tissue diseases.<br>Genetic disorders with metabolic or dysplastic skeletal abnormalities.<br>Organ transplantation.<br>Prolonged bed rest or immobilization (>3 months).<br>Cerebral palsy, muscular dystrophy, spinal muscular atrophy.
2. Limited to postmenopausal women<br>a. Maternal family history of osteoporotic fracture before the age of 75 years.<br>b. Menopause before the age of 45 years.<br>c. Low body weight: body mass index <19 kg/m^2.
**Minor risk factors** (at least three of the following)
1. Postmenopausal women<br>a. Age >65 years<br>b. Family history of severe osteoporosis<br>c. Premenopausal amenorrhea lasting more than 6 months<br>d. Inadequate calcium intake (<1200 mg/day)<br>e. Smoking >20 cigarettes/day<br>f. Alcohol abuse (>60 g/day of alcohol)
2. Men aged >60 years<br>a. Family history of severe osteoporosis<br>b. Low body weight (body mass index <19 kg/m^2)<br>c. Inadequate calcium intake (<1200 mg/day)<br>d. Smoking >20 cigarettes/day<br>e. Alcohol abuse (>60 g/day of alcohol)

**Table S3. Clinical characteristics of patients according to anti-osteoporosis treatment prior to the index hip fracture in the overall cohort.**				
**Variables**	**Total** ***(n=1103)***	**Untreated** ***(n=1021)***	**Treated** ***(n=82)***	**p**
Age (years), mean (SD)	82.8 (9.1)	82.9 (9.2)	80.8 (7.5)	**0.039**
Female sex, n (%)	790 (71.6)	715 (70.0)	75 (91.5)	**<0.001**
BMI (Kg/m^2), mean (SD)	24.1 (4.4)	24.1 (4.4)	23.5 (4.4)	0.246
Comorbidities				
Diabetes mellitus, n (%)	191 (17.3)	187 (18.3)	4 (4.9)	**0.002**
History of myocardial infarction, n (%)	124 (11.2)	121 (11.9)	3 (3.7)	**0.024**
Heart failure, n (%)	111 (10.1)	105 (10.3)	6 (7.3)	0.390
History of stroke, n (%)	98 (8.9)	93 (9.1)	5 (6.1)	0.357
COPD, n (%)	78 (7.1)	72 (7.1)	6 (7.3)	0.928
CKD stage IV-V, n (%)	43 (3.9)	40 (3.9)	3 (3.7)	0.907
Liver cirrhosis, n (%)	11 (1.0)	8 (0.8)	3 (3.7)	**0.012**
Rheumatologic diseases, n (%)	56 (5.1)	44 (4.3)	12 (14.6)	**<0.001**
Dementia, n (%)	260 (23.6)	246 (24.1)	14 (17.1)	0.150
CCI, median (IQR)	5 (4-6)	5 (4-6)	5 (4-6)	0.483
Risk factors for skeletal fragility				
Previous fracture, n (%)	335 (30.4)	298 (29.2)	37 (45.1)	**0.004**
Previous MOF, n (%)	241 (21.8)	213 (20.9)	28 (34.1)	**0.008**
Previous HF, n (%)	63 (5.7)	60 (5.9)	3 (3.7)	0.558
Previous vertebral fracture, n (%)	110 (10.0)	90 (8.8)	20 (24.4)	**<0.001**
Previous humerus fracture, n (%)	42 (3.8)	38 (3.7)	4 (4.9)	0.821

Previous wrist fracture, n (%)	70 (6.3)	65 (6.4)	5 (6.1)	1.000
Previous fracture at another site, n (%)	149 (13.5)	137 (13.4)	12 (14.6)	0.887
Family history of hip fracture, n (%)	110 (10.0)	102 (10.0)	8 (9.8)	1.000
Active smoking, n (%)	83 (7.5)	75 (7.4)	8 (9.8)	0.565
Chronic glucocorticoid therapy, n (%)	26 (2.4)	17 (1.7)	9 (11.0)	**<0.001**
FRAX® MOF, median (IQR)†	19.0 (10.0-26.0)	19.0 (10.0-25.0)	22.0 (16.8-32.2)	**<0.001**
FRAX® HF, median (IQR)†	9.9 (5.6-15.0)	9.7 (5.3-15.0)	11.5 (8.3-19.0)	**0.001**
DeFRA, median (IQR)‡	20.0 (15.0-32.8)	19.0 (14.0-31.0)	29.0 (18.9-52.2)	**<0.001**

†Referring only to subjects aged 50-90 years. ‡Referring only to female subjects aged 50-90 years. Statistically significant p-values (p<0.05) are highlighted in bold.

Abbreviations: BMI: body mass index, CCI: Charlson Comorbidity Index, CKD: chronic kidney disease, COPD: chronic obstructive pulmonary disease, DeFRA: Derived Fracture Risk Assessment Tool, FRAX®: Fracture Risk Assessment Tool, HF: hip fracture, IQR: interquartile range, MOF: major osteoporotic fracture, SD: standard deviation.

**Table S4. Univariable and multivariable logistic regression analyses of factors associated with anti-osteoporosis treatment prior to the index hip fracture among female subjects.**				
**Variables**	**Univariable**		**Multivariable**	
	**OR (95% CI)**	**p**	**OR (95% CI)**	**p**
Age (years)	0.97 (0.94–0.99)	**0.005**	0.98 (0.95–1.00)	0.060
BMI (Kg/m^2)	0.99 (0.93–1.05)	0.701	–	–
Comorbidities				
Diabetes mellitus	0.28 (0.10–0.78)	**0.015**	0.28 (0.10–0.81)	**0.019**
History of myocardial infarction	0.30 (0.07–1.25)	0.098	0.38 (0.09–1.62)	0.190
Heart failure	0.84 (0.35–2.01)	0.697	–	–
History of stroke	0.73 (0.26–2.09)	0.562	–	–
COPD	2.06 (0.83–5.13)	0.122	–	–
CKD stage IV-V	1.31 (0.38–4.49)	0.665	–	–
Liver cirrhosis	29.75 (3.05–289.73)	**0.003**	–	–
Rheumatologic diseases	3.94 (1.94–8.00)	**<0.001**	3.34 (1.54–7.25)	**0.002**
Dementia	0.68 (0.37–1.27)	0.229	–	–
CCI	1.00 (0.88–1.14)	0.967	–	–
Risk factors for skeletal fragility				
Previous fracture	1.86 (1.15–3.00)	**0.012**	1.79 (1.08–2.95)	**0.024**
Previous MOF	1.71 (1.03–2.84)	**0.037**	–	–
Previous HF	0.41 (0.10–1.72)	0.221	–	–
Previous vertebral fracture	3.28 (1.84–5.85)	**<0.001**	–	–
Previous humerus fracture	0.86 (0.26–2.88)	0.808	–	–

Previous wrist fracture	0.84 (0.33–2.17)	0.719	–	–
Previous fracture at another site	1.12 (0.58–2.15)	0.735	–	–
Family history of hip fracture	1.14 (0.52–2.46)	0.747	–	–
Active smoking	1.83 (0.79–4.26)	0.159	–	–
Chronic glucocorticoid therapy	9.61 (3.77–24.49)	**<0.001**	5.98 (2.18–16.39)	**<0.001**
FRAX® MOF†	1.02 (1.00–1.05)	**0.023**	–	–
FRAX® HF†	1.02 (1.00–1.04)	**0.047**	–	–
DeFRA‡	1.02 (1.01–1.03)	**<0.001**	–	–

†Referring only to subjects aged 50-90 years. ‡Referring only to female subjects aged 50-90 years. Statistically significant p-values ($p<0.05$) are highlighted in bold.

Abbreviations: BMI: body mass index, CCI: Charlson Comorbidity Index, CKD: chronic kidney disease, CI: confidence interval, COPD: chronic obstructive pulmonary disease, DeFRA: Derived Fracture Risk Assessment Tool, FRAX®: Fracture Risk Assessment Tool, HF: hip fracture, MOF: major osteoporotic fracture, OR: odds ratio.

Table S5. Clinical characteristics of patients according to FRAX® risk category prior to the index fracture.

Variables	Low risk (n=58)	Intermediate risk (n=190)	High risk (n=88)	Very high risk (n=481)	p overall	p†
Age (years), mean (SD)	67.4 (6.5)	76.3 (9.5)	81.4 (6.2)	83.3 (5.9)	**<0.001**	**<0.001**
Female sex, n (%)	18 (31.0)	76 (40.0)	48 (54.5)	428 (89.0)	**<0.001**	**<0.001**
BMI (Kg/m^2), mean (SD)	27.8 (5.4)	25.8 (4.6)	26.1 (4.5)	22.9 (3.7)	**<0.001**	**<0.001**
Comorbidities						
Diabetes mellitus, n (%)	12 (20.7)	40 (21.1)	13 (14.8)	79 (16.4)	0.410	0.120
History of myocardial infarction, n (%)	9 (15.5)	29 (15.3)	7 (8.0)	49 (10.2)	0.141	**0.033**
Heart failure, n (%)	5 (8.6)	15 (7.9)	5 (5.7)	49 (10.2)	0.515	0.603
History of stroke, n (%)	5 (8.6)	16 (8.4)	12 (13.6)	42 (8.7)	0.500	0.739
COPD, n (%)	4 (6.9)	25 (13.2)	11 (12.5)	29 (6.0)	**0.011**	**0.039**
CKD stage IV-V, n (%)	1 (1.7)	3 (1.6)	2 (2.3)	21 (4.4)	0.239	0.116
Liver cirrhosis, n (%)	1 (1.7)	1 (0.5)	1 (1.1)	7 (1.5)	0.776	0.711
Rheumatologic diseases, n (%)	5 (8.6)	7 (3.7)	2 (2.3)	32 (6.7)	0.162	0.629
Dementia, n (%)	6 (10.3)	38 (20.0)	20 (22.7)	111 (23.1)	0.149	0.110
CCI, median (IQR)	4 (2-5)	5 (3-6)	5 (4-6)	5 (4-6)	**<0.001**	**<0.001**
Risk factors for skeletal fragility						
Previous fracture, n (%)	2 (3.4)	24 (12.6)	28 (31.8)	191 (39.7)	**<0.001**	**<0.001**
Previous MOF, n (%)	2 (3.4)	17 (8.9)	20 (22.7)	136 (28.3)	**<0.001**	**<0.001**
Previous HF, n (%)	1 (1.7)	2 (1.1)	4 (4.5)	38 (7.9)	**0.003**	**0.001**
Previous vertebral fracture, n (%)	0 (0.0)	10 (5.3)	9 (10.2)	62 (12.9)	**0.001**	**<0.001**

Previous humerus fracture, n (%)	1 (1.7)	1 (0.5)	4 (4.5)	21 (4.4)	0.067	**0.015**
Previous wrist fracture, n (%)	0 (0.0)	7 (3.7)	6 (6.8)	40 (8.3)	**0.027**	**0.008**
Previous fracture at another site, n (%)	1 (1.7)	10 (5.3)	15 (17.0)	85 (17.7)	**<0.001**	**<0.001**
Family history of hip fracture, n (%)	4 (6.9)	14 (7.4)	4 (4.5)	72 (15.0)	**0.003**	**0.017**
Active smoking, n (%)	6 (10.3)	21 (11.1)	9 (10.2)	34 (7.1)	0.331	0.153
Chronic glucocorticoid therapy, n (%)	0 (0.0)	5 (2.6)	1 (1.1)	17 (3.5)	0.318	0.496
FRAX® MOF, median (IQR)	5.0 (3.8-5.7)	9.4 (8.0-11.0)	15.0 (11.0-17.0)	24.0 (21.0-32.0)	**<0.001**	**<0.001**
FRAX® HF, median (IQR)	1.4 (0.9-2.0)	4.7 (3.5-5.7)	7.3 (6.9-8.0)	14.0 (11.0-20.0)	**<0.001**	**<0.001**
DeFRA, median (IQR)*	7.0 (5.0-12.5)	11.0 (8.0-14.0)	14.0 (13.0-19.2)	23.0 (18.0-43.2)	**<0.001**	**<0.001**
Treatments before index fracture						
Vitamin D supplements, n (%)	8 (13.8)	54 (28.4)	24 (27.3)	201 (41.8)	**<0.001**	**<0.001**
Anti-osteoporosis treatment, n (%)	1 (1.7)	9 (4.7)	8 (9.1)	50 (10.4)	**0.026**	**0.005**

†Comparison between low/intermediate vs. high/very high FRAX risk groups. Statistically significant p-values ($p<0.05$) are highlighted in bold.

Abbreviations: BMI: body mass index, CCI: Charlson Comorbidity Index, CKD: chronic kidney disease, COPD: chronic obstructive pulmonary disease, DeFRA: Derived Fracture Risk Assessment Tool, FRAX®: Fracture Risk Assessment Tool, HF: hip fracture, IQR: interquartile range, MOF: major osteoporotic fracture, SD: standard deviation.
